# Finite-Stroke Magnetic Quasi-Zero-Stiffness Electromagnetic Harvester for Foot-Worn Sensors: Reproducible Numerical Design Under Public Foot-IMU Excitation

**DOI:** 10.3390/mi17080892

**Published:** 2026-07-25

**Authors:** Mohamed Hamdaoui

**Affiliations:** Université de Lorraine, UFR de Mathématiques, Informatique et Mécanique (UFR MIM), LEM3 UMR CNRS 7239, 7 Rue Félix Savart, 57070 Metz, France; mohamed.hamdaoui@univ-lorraine.fr

**Keywords:** electromagnetic energy harvesting, quasi-zero stiffness, finite stroke, foot-mounted IMU, position-dependent flux linkage, sectioned coil, soft end stop, reproducible numerical design

## Abstract

Foot-worn electromagnetic harvesters are driven by irregular rigid-body motion, while their response is limited by mechanical stroke, coil geometry, mounting direction, and the electrical interface. This paper presents a reproducible numerical design study of a finite-stroke magnetic quasi-zero-stiffness (QZS) moving-magnet harvester. Two public three-axis foot-IMU records are processed with stated gyroscope-bias estimation, six-axis attitude estimation, gravity removal, residual-offset correction, filtering, and angular-acceleration calculation. Three explicit axes are used in the design screen, and the selected candidate is then evaluated over a 62-direction spherical grid. Rigid-body angular-acceleration and centripetal terms are included for specified sensor-to-harvester offsets. Two normalized magnetic force laws are compared. The electrical model uses position-dependent flux linkage, explicit series connection and polarity of coil sections, winding-derived resistance, and a position-dependent electromagnetic reaction force. A fixed-seed random screen evaluates 720 geometry-constrained candidates. The highest-ranked nominal candidate is a 150 mm external foot-worn module with a 40.6 g moving mass, a 30 mm hard half-stroke, 1649 turns in two series sections, and a 25.27 mm coil outer diameter. Across 72 design-screen cases formed from 12 five-second windows, three mounting axes, and two magnetic laws, this candidate remained hard-stroke- and design-stroke-safe. Its conditional ideal load-side power had a 10th percentile of 1.38 mW and a median of 2.03 mW. In the 62-direction check, all 1488 cases remained hard-stroke-safe; two opposite directions each produced one design-stroke exceedance, with a maximum displacement of 24.15 mm. Re-ranking all 30 Stage-2 candidates under coupling and magnetic-stiffness changes retained the long geometry family, although a 30% coupling reduction changed the highest-ranked candidate from 600 to 632. Soft-stop sensitivity, equation-level consistency, and multi-case Runge–Kutta convergence are also reported. The results support finite-stroke design screening, but they do not constitute prototype, finite-element, or delivered-power validation.

## 1. Introduction

Wearable and foot-worn sensors require compact power sources. Energy harvesting can reduce battery replacement when the sensing task is intermittent and the available mechanical motion is sufficient [1,2,3,4]. Human walking is an attractive source, but it differs from a controlled sinusoidal input. Foot motion contains impact, swing, rotation, turns, and pauses. The installed device must also satisfy limits on length, mass, clearance, attachment, and moving travel.

Footwear harvesters have used piezoelectric, triboelectric, electromagnetic, and hybrid transduction. Experimental studies have demonstrated shoe-embedded piezoelectric devices [5,6,7], sole-bending electromagnetic conversion [8], and larger biomechanical harvesters at body joints [9]. Recent reviews summarize the broader wearable field and its system-level constraints [10,11,12,13]. These studies show that reported power cannot be separated from device size, excitation, load, and test method.

A moving-magnet electromagnetic harvester is mechanically simple because the coil can remain fixed. Its main limitation is finite relative travel. A low natural frequency can increase response under walking excitation, but a numerical response is not useful if the shuttle crosses the device stroke. Quasi-zero-stiffness (QZS) mechanisms reduce the local restoring stiffness by combining positive and negative stiffness. QZS concepts are established in vibration isolation and energy harvesting [14,15,16]. For a foot-worn device, however, low local stiffness increases sensitivity to friction, magnetic alignment, mounting orientation, and end-stop engagement.

The present work develops and evaluates a numerical model for finite-stroke design selection. Its purpose is to determine which conclusions remain supported when the excitation, magnetic force law, electromagnetic coupling, design search, and soft-stop model are stated in reproducible form. Table 1 positions the work against representative studies. The comparison is qualitative because the test conditions differ.

The study makes five contributions. First, it replaces scalar acceleration and gyroscope projection factors with full three-axis preprocessing, explicit sensor-to-harvester axis vectors, and a 62-direction post-selection orientation sweep. Second, it compares two magnetic force shapes with the same local negative stiffness. Third, it replaces constant electromagnetic coupling with a position-dependent flux-linkage model for series-connected coil sections with explicit winding polarity. Fourth, it reports the fixed-seed design-screen procedure, bounds, constraints, ranking rule, evaluated-design count, and full-candidate uncertainty re-ranking. Fifth, it separates nominal stop-free operation from a controlled stop-engagement stress case and reports multi-case solver convergence.

The evidence boundary is important. The public records do not provide a calibrated transformation for a proposed harvester, and the magnetic dimensions, remanence, force–displacement curve, and measured flux linkage are not available. The mounting cases and coupling scale are therefore stated assumptions. The numerical results are conditional on those assumptions and are not presented as measured device performance.

## 2. Device and Governing Model

### 2.1. Mechanical Layout

**Figure 1:**
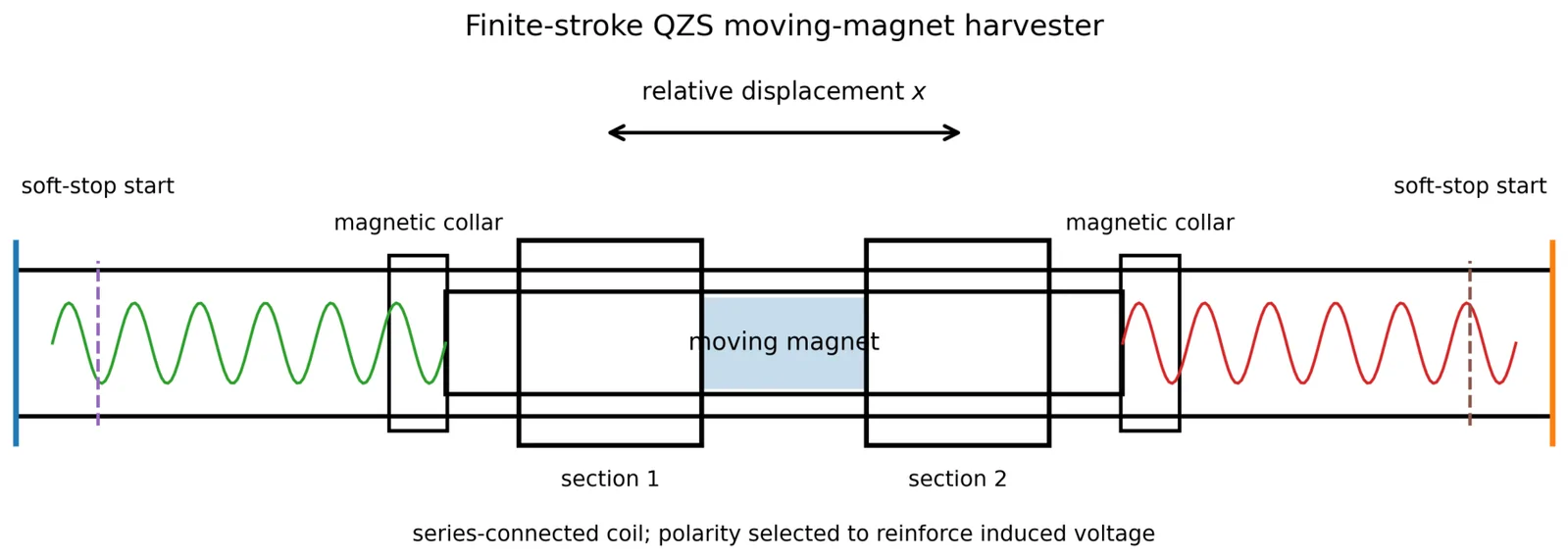
Modeled finite-stroke QZS moving-magnet harvester and two-section coil topology.

Figure 1 shows the modeled architecture. A magnet-containing shuttle moves along a guide tube. Positive stiffness is supplied by two preloaded springs. Fixed magnetic elements provide local negative stiffness. The coil is fixed to the housing and may be divided into series-connected sections. Cubic soft stops engage before the hard travel limit. The long candidate is intended as an external lateral shoe-rail or ankle-brace module. It is not an under-insole design.

Let *x* be shuttle displacement relative to the housing, positive along the harvester axis. The single-axis equation used in the design screen is

(1) $$m\ddot{x}+c_{m}\dot{x}+k_{p}x+F_{m}\left(x\right)+F_{\mathrm{stop}}\left(x,\dot{x}\right)+c_{e}\left(x\right)\dot{x}-m\omega_{⊥}^{2}\left(t\right)x=-ma_{b}\left(t\right),$$

where *m* is moving mass, $c_{m}$ is mechanical viscous damping, $k_{p}$ is total positive stiffness, $F_{m}$ is the negative-stiffness magnetic force, $c_{e}\left(x\right)$ is position-dependent electromagnetic damping, $a_{b}$ is housing acceleration projected along the harvester axis, and $\omega_{⊥}$ is the angular-rate component perpendicular to that axis. The term $-m\omega_{⊥}^{2}x$ is the axial component of the rotating-frame centripetal term for relative motion. Coriolis terms vanish in this one-degree-of-freedom formulation because both relative displacement and velocity are constrained to the harvester axis.

Equation (1) is a screening model. It does not include lateral shuttle motion, Coulomb friction, guide clearance, magnetic side load, structural flexibility, acoustic response, or wear.

### 2.2. Rigid-Body Excitation and Mounting Transformation

The source files contain three-axis accelerometer and gyroscope records from a foot-mounted IMU. Let *S* be the sensor origin and *H* the harvester reference point. For a rigid attachment with constant vector $r_{H/S}$ expressed in the sensor frame, the gravity-compensated linear acceleration at *H* is

(2) $$a_{H}=a_{S}+\alpha \times r_{H/S}+\omega \times \left(\omega \times r_{H/S}\right),$$

where $a_{S}$ is gravity-compensated sensor acceleration, ω is angular rate, and α=dω/dt is angular acceleration. If $e_{h}$ is a unit vector along the harvester axis, expressed in the sensor frame, then

(3) $$a_{b}=e_{h}^{T}a_{H},\;\omega_{⊥}^{2}={∥\omega ∥}^{2}-{\left(e_{h}^{T}\omega \right)}^{2}.$$

In a calibrated installation, $e_{h}$ would be obtained from a measured sensor-to-harvester rotation matrix. Such a calibration is not available for the public records. Three explicit bounded cases are therefore used instead of fitted scalar factors:

(4) $$e_{X}={\left[\begin{array}{ccc} 1 & 0 & 0 \end{array}\right]}^{T},\;e_{Y}={\left[\begin{array}{ccc} 0 & 1 & 0 \end{array}\right]}^{T},\;e_{XZ30}={\left[\begin{array}{ccc} \cos30^{\circ } & 0 & \sin30^{\circ } \end{array}\right]}^{T}.$$

The main design screen uses a co-located sensor and harvester reference, $r_{H/S}=0$. A separate check evaluates 20 mm offsets along positive sensor *X*, positive sensor *Y*, and both directions of sensor *Z*.

The three axes in Equation (4) keep the candidate screen computationally bounded. After selection, orientation is examined without fitted projection factors using the deterministic spherical grid

(5) $$e_{h}\left(\beta ,\psi \right)={\left[\begin{array}{ccc} \sin\beta \cos\psi & \sin\beta \sin\psi & \cos\beta \end{array}\right]}^{T}.$$

The polar angles are $0^{\circ },30^{\circ },60^{\circ },90^{\circ },120^{\circ },150^{\circ },180^{\circ }$. For each intermediate polar angle, the azimuth is sampled every $30^{\circ }$; one direction is used at each pole. This gives 62 unit vectors. The selected candidate is evaluated for all 12 windows and both magnetic laws at each direction, giving 1488 post-selection orientation cases. This sweep is a bounded numerical orientation study, not a calibrated reconstruction of the public sensor installation.

### 2.3. Magnetic Force Laws

The first magnetic force law is the rational negative-stiffness surrogate

(6) $$F_{m}^{\left(R\right)}\left(x\right)=-\frac{k_{m}x}{1+{\left(x/x_{m}\right)}^{2}},$$

where $k_{m}$ is the magnitude of the local negative stiffness and $x_{m}$ controls force roll-off. Its local slope is ${\left.dF_{m}^{\left(R\right)}/dx\right|}_{0}=-k_{m}$.

An independent analytical model-form check uses the axial gradient of a dipole-interaction potential with a fixed transverse separation [19]:

(7) $$F_{m}^{\left(D\right)}\left(x\right)=-\frac{k_{m}x}{{\left[1+{\left(x/x_{m}\right)}^{2}\right]}^{5/2}}.$$

This expression is normalized to the same local slope, $-k_{m}$. It tests whether the selected design depends on the force-law shape. It is not a finite-element representation of the proposed collars because collar geometry, magnet dimensions, remanence, permeability, and alignment tolerances are not specified.

Before stop engagement, the zero-speed local stiffness at equilibrium is

(8) $$k_{\mathrm{eff}}=k_{p}-k_{m}.$$

The screen restricts $k_{m}/k_{p}$ to 0.72–0.92, so $k_{\mathrm{eff}}>0$ for every generated candidate.

### 2.4. Soft-Stop Model

The soft stop begins at $\left|x\right|=x_{s}<x_{\mathrm{hard}}$. With penetration $\delta =\left|x\right|-x_{s}$ and inward penetration speed $\dot{\delta }=\mathrm{sgn}\left(x\right)\dot{x}$, the force is

(9) $$F_{\mathrm{stop}}\left(x,\dot{x}\right)=\left\{\begin{array}{cc} 0, & \left|x\right|\leq x_{s},\\ \mathrm{sgn}\left(x\right)\left[k_{s}\delta^{3}+c_{s}\max\left(0,\dot{\delta }\right)\right], & |x|>x_{s}. \end{array}\right.$$

The cubic coefficient is calculated from a sampled elastic force at the hard limit,

(10) $$k_{s}=\frac{F_{s,\mathrm{hard}}}{{\left(x_{\mathrm{hard}}-x_{s}\right)}^{3}}.$$

This parameterization states the force scale directly. Stop damping acts only during deeper penetration and is zero during rebound. The stop values are numerical design variables, not measured bumper properties.

## 3. Position-Dependent Electromagnetic Model

### 3.1. Flux Linkage and Coil-Section Polarity

For an axial magnetic dipole and a circular loop of radius *a*, the flux shape per turn is proportional to [19]

(11) $$\phi \left(z\right)=\frac{a^{2}}{2{\left(a^{2}+z^{2}\right)}^{3/2}}.$$

For section *j* with $N_{j}$ turns, winding sign $s_{j}\in \left\{-1,+1\right\}$, section-center coordinate $z_{j}$, and uniformly distributed turns over length $L_{j}$, the model uses

(12) $$\lambda \left(x\right)=C_{\lambda }\sum_{j=1}^{n_{s}}s_{j}N_{j}{\langle \phi (z-x)\rangle }_{z\in L_{j}},$$

where $C_{\lambda }$ contains the omitted magnet dipole moment and magnetic constant. The position-dependent coupling is

(13) $$\kappa \left(x\right)=\frac{d\lambda }{dx}.$$

The magnet dimensions and remanence are unavailable. The absolute scale is therefore conditional: $C_{\lambda }$ is selected so that the root-mean-square value of κ(x) over the design stroke equals

(14) $$\kappa_{\mathrm{ref}}=0.85\left(\frac{N}{700}\right)\left(\frac{10.8\;\mathrm{mm}}{\bar{r}}\right)\;V\;s\;m^{-1},$$

where *N* is total turn count and $\bar{r}$ is mean winding radius. Equation (14) retains the baseline geometry scale while adding the analytical spatial variation. It is not an experimentally identified coupling law. A ±30% scale variation is included in the uncertainty study.

All sections are connected in series. The total resistance is

(15) $$R_{c}=\sum_{j=1}^{n_{s}}R_{c,j}.$$

The first section sign is fixed at +1 and the remaining signs are chosen from all $2^{n_{s}-1}$ combinations to maximize the root-mean-square unscaled κ(x) over the design stroke. This avoids voltage cancellation caused by using the same winding direction for sections on opposite sides of the equilibrium position.

With the sign convention $e=-\kappa \left(x\right)\dot{x}$, a purely resistive load gives

(16) $$i=-\frac{\kappa \left(x\right)\dot{x}}{R_{c}+R_{L}},\;P_{L}=i^{2}R_{L},$$

and the electromagnetic reaction force is

(17) $$F_{e}=\kappa \left(x\right)i=-\frac{\kappa^{2}\left(x\right)}{R_{c}+R_{L}}\dot{x}.$$

Thus, the damping coefficient in Equation (1) is $c_{e}\left(x\right)=\kappa^{2}\left(x\right)/\left(R_{c}+R_{L}\right)$. Coil inductance, rectification, converter start-up, storage, leakage, and sensor duty cycle are not included. $P_{L}$ is therefore an ideal AC load-side quantity.

### 3.2. Winding Geometry

The guide-tube outer diameter is 18 mm, radial winding clearance is 0.5 mm, and coil outer diameter is restricted to 26 mm. Five copper wire options are evaluated. The turn pitch is 1.08 times insulated wire diameter. For winding layer *q*,

(18) $$r_{q}=\frac{D_{t}}{2}+t_{c}+\left(q-\frac{1}{2}\right)d_{o}.$$

The wire length, mean radius, and resistance are

(19) $$L_{w}=\sum_{q}2\pi r_{q}N_{q}+L_{\mathrm{lead}},\;\bar{r}=\frac{\sum_{q}r_{q}N_{q}}{N},\;R_{c}=\rho_{\mathrm{Cu}}\frac{L_{w}}{\pi d_{\mathrm{Cu}}^{2}/4},$$

with $L_{\mathrm{lead}}=0.15$ m and $\rho_{\mathrm{Cu}}=1.724\times 10^{-8}\;\Omega \;m$.

## 4. IMU Records and Preprocessing

### 4.1. Source Records

The analysis uses the public short_walk.csv and long_walk.csv records from the x-io Technologies Gait-Tracking repository [20]. The repository identifies them as foot-mounted NGIMU example records and supplies the associated processing code. Both raw files and the complete MIT license are included with the source archive. No clinical cohort or population inference is made from these two example records.

### 4.2. Preprocessing Sequence

The complete sequence is as follows.

1. Duplicate timestamps are removed and the records are sorted by time.
2. Accelerometer and gyroscope channels are linearly interpolated to 400 Hz, matching the public example code [21].
3. Gyroscope bias is estimated with the Fusion stationary-bias algorithm using a $3\;\deg\;s^{-1}$ threshold and a 3 s stationary period.
4. A six-axis Fusion AHRS is run without magnetometer using the public example settings: NWU convention, gain 0.5, gyroscope range $2000\;\deg\;s^{-1}$, acceleration-rejection threshold $10^{\circ }$, and 5 s recovery period [21,22].
5. Sensor-frame linear acceleration is obtained by subtracting the AHRS gravity estimate from the accelerometer measurement. The result is multiplied by $g_{0}=9.80665\;m\;s^{-2}$.
6. An eighth-order zero-phase Butterworth low-pass filter with a 14 Hz cut-off is applied to linear acceleration and angular rate.
7. The mean residual acceleration and angular rate over the 3.5–7.0 s static interval are subtracted. This removes constant residual offsets; it is not a scale or alignment calibration.
8. Angular acceleration is calculated by centered numerical differentiation of the filtered angular rate and is filtered again at 14 Hz.
9. The processed signals are resampled to 100 Hz for the mechanical simulation.

Fusion may reject accelerometer feedback while dynamic acceleration is large. The reported “ignored” fraction refers only to the accelerometer correction used by the attitude estimator; no excitation samples are removed from the mechanical input. Table 2 reports the preprocessing outputs.

**Figure 2:**
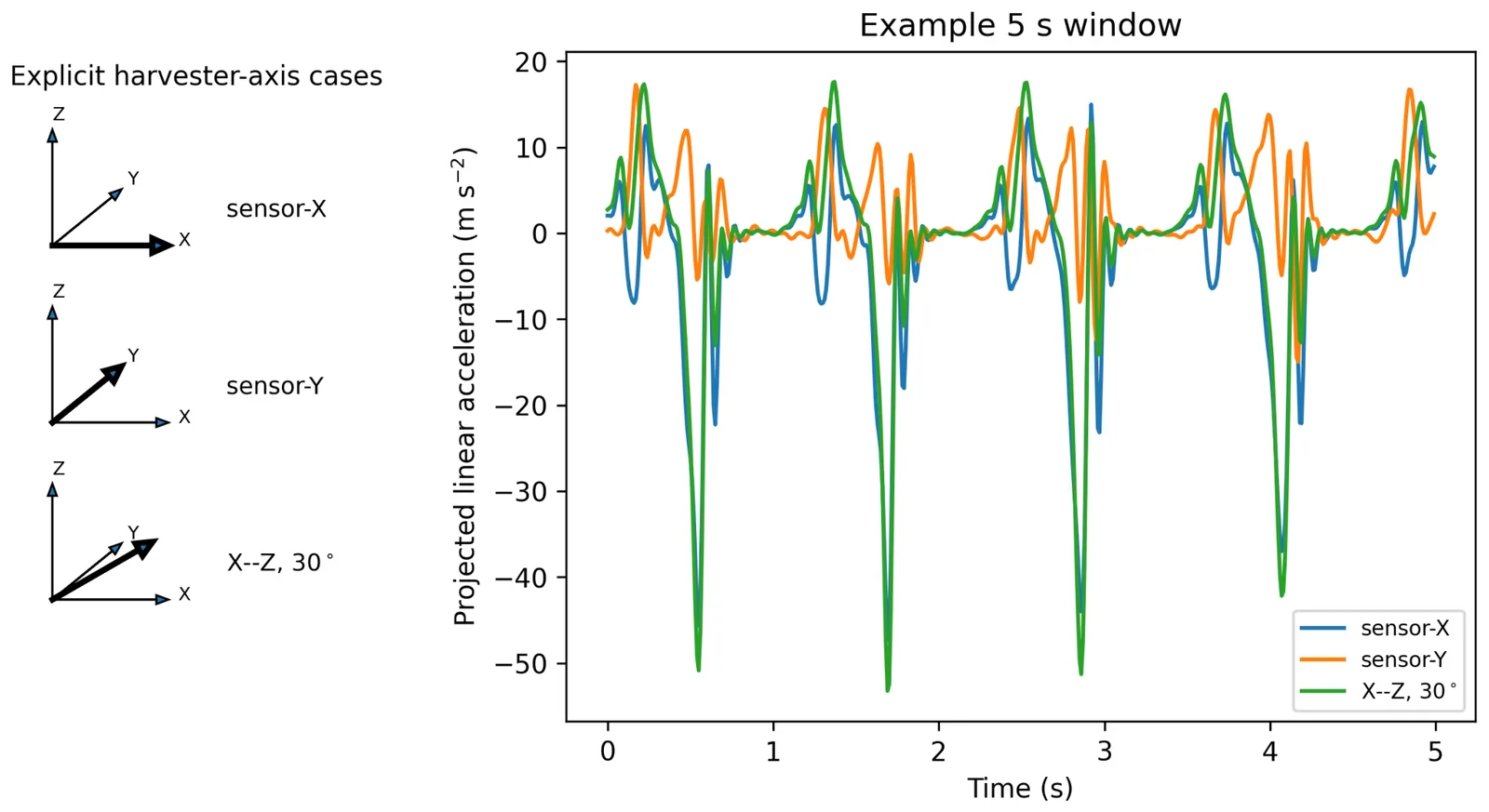
Explicit harvester-axis cases and projected gravity-compensated acceleration for the 40–45 s long-record window.

Twelve non-overlapping five-second windows are used: four from the short record and eight from the long record. Their exact start and end times are listed in the Supplementary Materials. Figure 2 shows the three axis definitions and the projected acceleration for one window. The differences are generated by the vector projection itself; no scalar amplitude factors are fitted.

## 5. Reproducible Design Screen

### 5.1. Candidate Generation and Geometry Families

The design procedure is a fixed-seed random screen, not a proof of a global optimum. The NumPy pseudorandom generator seed is 4,393,728. Three device families are used:

- Compact: length 90 mm, hard/design half-stroke 15/12 mm;
- Medium: length 120 mm, hard/design half-stroke 22/17.6 mm;
- Long: length 150 mm, hard/design half-stroke 30/24 mm.

For each family, 240 feasible winding/mechanical candidates are generated, giving 720 candidates. Table 3 lists the common bounds. Family-specific turn count, coil length, and section-count bounds are provided in the Supplementary Materials.

A winding candidate is rejected during generation if the requested turn count cannot fit within the selected coil length and the 26 mm outer-diameter limit. No candidate is rejected on power. Generated candidates all have positive local equilibrium stiffness because $k_{m}/k_{p}<1$.

### 5.2. Two-Stage Evaluation and Ranking

Stage 1 evaluates every candidate on four fixed five-second windows using the sensor-*X* axis, the rational magnetic law, and three internal fourth-order Runge–Kutta (RK4) substeps per 10 ms input interval. Each family is ranked and its first ten candidates are retained.

Stage 2 evaluates the resulting 30 candidates on 12 windows, three mounting axes, and two magnetic laws. This gives 72 cases per candidate and 2160 Stage-2 simulations. Four RK4 substeps per 10 ms input interval are used. Candidates are sorted lexicographically by:

1. Decreasing hard-stroke-safe fraction;
2. Decreasing design-stroke-safe fraction;
3. Decreasing 10th-percentile ideal load power;
4. Decreasing median ideal load power;
5. Increasing 95th-percentile peak displacement.

The 0.05 mW threshold is not used in candidate generation, constraint handling, or ranking. It is evaluated only in a separate reporting-threshold sweep.

The hard-safe condition is $\max|x|\leq x_{\mathrm{hard}}$. The design-safe condition is $\max|x|\leq x_{\mathrm{design}}$. A run is marked numerically unstable if |x|>0.5 m or a non-finite state occurs. Complete candidate parameters, Stage-1 results, Stage-2 cases, and the ranking table are included in the source archive.

To test whether parameter uncertainty changes the selected design, all 30 Stage-2 candidates are re-evaluated and re-ranked under four global changes: coupling scale 0.7× and 1.3× and magnetic stiffness 0.8× and 1.2×. Each scenario uses the same 72 cases and the same lexicographic rule. This adds 30×72×4=8640 simulations. The uncertainty re-ranking changes model parameters only; the candidate geometry and load resistance are not re-optimized within a scenario.

## 6. Results

### 6.1. Magnetic and Electromagnetic Model Checks

**Figure 3:**
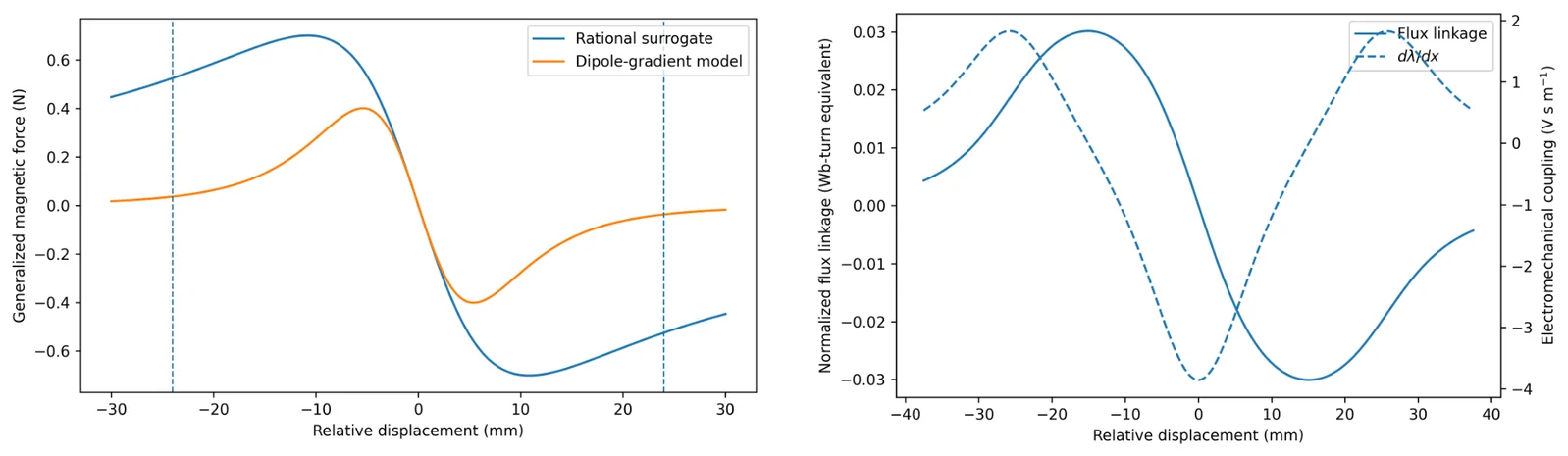
Normalized magnetic force laws (**left**) and position-dependent flux linkage and coupling for the selected two-section coil (**right**). Dashed vertical lines on the force plot indicate the design half-stroke.

Figure 3 compares the two magnetic force laws and the selected coil’s flux-linkage and coupling profiles. Both magnetic laws have the same local slope. Their large-displacement roll-off differs. The two-section flux linkage is not monotonic, so κ(x) changes magnitude and sign as the magnet traverses the coil. Power and reaction-force calculations use the local value of κ(x).

The selected two-section coil uses opposite section signs, written +/−. Over the design stroke, its unscaled root-mean-square coupling is used as the reference value. The same-sign +/+ connection gives 0.473 times that value. Thus the model predicts substantial voltage cancellation if both sections are wound or connected with the same polarity.

Three equation-level implementation checks were performed. First, a centered finite-difference derivative of the flux-linkage array on a 10,001-point grid agreed with the analytical coupling array to within $2.6\times 10^{-5}\%$ of peak coupling. Second, the numerical slopes of both magnetic laws at equilibrium agreed with the prescribed value $-k_{m}$ to within $2.2\times 10^{-10}\%$. Third, the maximum instantaneous mismatch between the mechanical power removed by $F_{e}$ and the total resistive power was $2.78\times 10^{-17}$ W in a representative case. These checks verify the numerical implementation of the stated equations; they do not validate the force or coupling magnitude against hardware.

### 6.2. Selected Candidate and Family Comparison

Table 4 gives the highest-ranked evaluated candidate. It belongs to the long family and is candidate 600 in the archived parameter table. The local stiffness cancellation is 72.84%, leaving 48.27 N/m positive equilibrium stiffness. The moving mass is 40.57 g. Total installed device mass was not modeled and is not reported.

Across its 72 Stage-2 cases, the selected candidate remained inside both the 30 mm hard half-stroke and 24 mm design half-stroke. The median stop-contact fraction was zero. The 10th-percentile, median, and 90th-percentile conditional ideal load powers were 1.385, 2.027, and 3.196 mW. The median, 95th-percentile, and maximum peak displacements were 14.34, 18.95, and 21.22 mm. The corresponding median RMS load voltage was 0.296 V.

The response depends on mounting direction. Across both magnetic laws and all 12 windows, the median powers were 3.005 mW for sensor *X*, 1.644 mW for sensor *Y*, and 1.911 mW for the *X*–*Z* 30^∘^ case. The maximum peak displacements were 21.22, 13.72, and 18.90 mm, respectively. These differences support treating installation orientation as a design input rather than a fitted projection coefficient.

**Figure 4:**
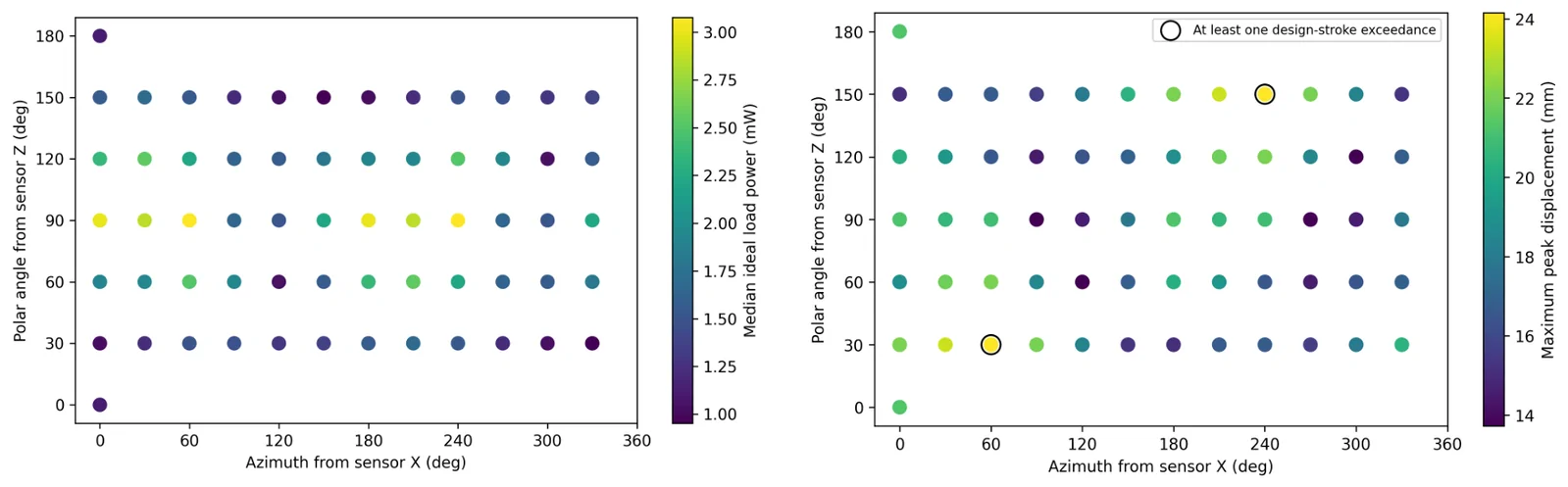
Selected-candidate response over the 62-direction spherical grid: median ideal load power (**left**) and maximum peak displacement (**right**). Each direction contains 12 windows and two magnetic laws.

The 62-direction post-selection sweep gives the broader directional result shown in Figure 4. All 1488 cases remain inside the 30 mm hard half-stroke. Sixty directions are design-safe in all 24 window–model combinations. Two opposite directions each have one design-stroke exceedance; the largest peak displacement is 24.15 mm. Direction-level median ideal power ranges from 0.952 to 3.077 mW. The sweep shows that the hard-stroke conclusion is robust over the sampled sphere, whereas the 24 mm design margin is sensitive to a small set of orientations.

**Figure 5:**
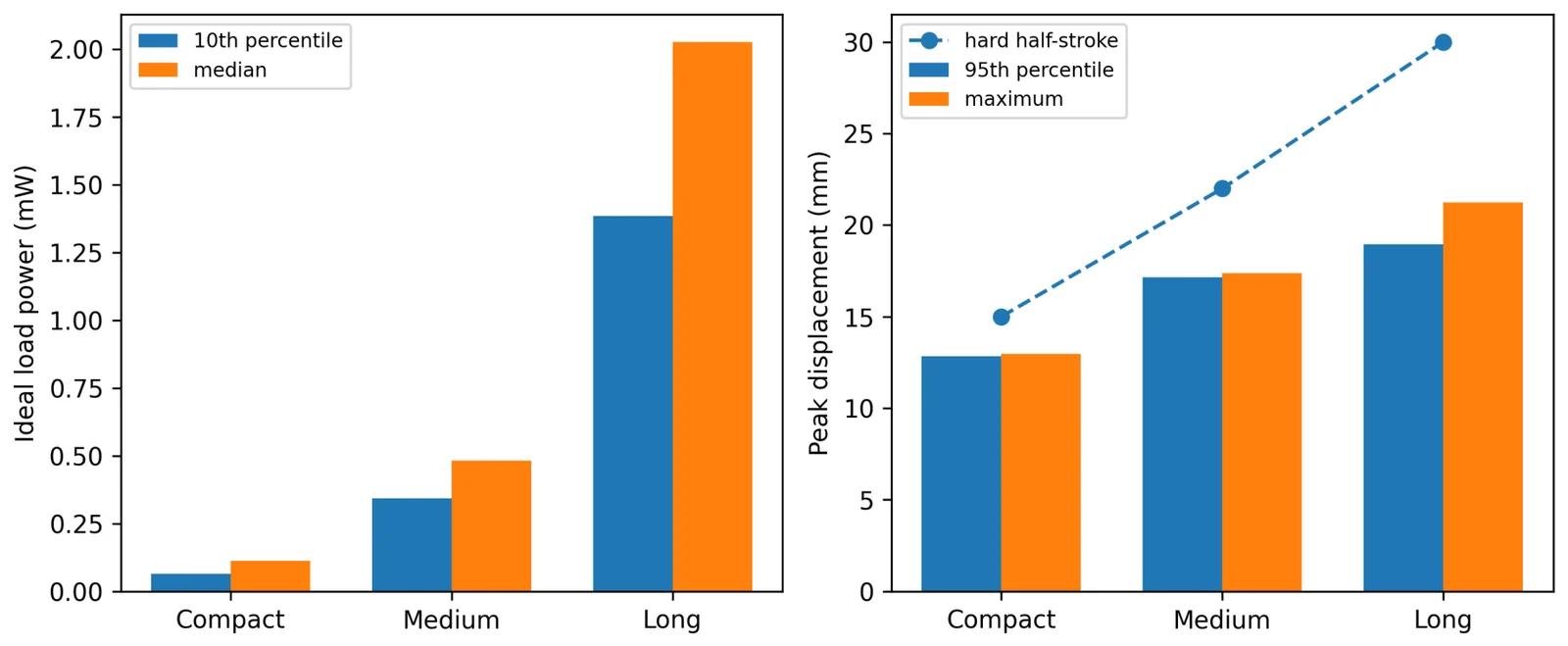
Power and displacement metrics for the highest-ranked Stage-2 candidate in each geometry family.

Figure 5 compares the highest-ranked Stage-2 candidate from each family. The compact candidate remained hard-safe but was design-safe in only 52.8% of its 72 cases. The medium and long candidates were hard- and design-safe in all the evaluated cases. The long candidate produced the largest lower-tail and median ideal load power among the evaluated families.

The selected long candidate was stable in the Stage-1 prefix check from 120 through 240 long-family candidates. This check does not prove convergence to a global optimum; it shows that the selected identifier did not change when the fixed random sequence was extended over that range.

### 6.3. Magnetic-Law Ranking and Parameter Uncertainty

When the Stage-2 cases are ranked separately by magnetic law, candidate 600 ranks first under both laws. Under the rational law, the selected candidate has median power 2.106 mW and 95th-percentile peak displacement 20.04 mm. Under the dipole-gradient law, the corresponding values are 1.984 mW and 17.14 mm. Both sets remain hard- and design-safe.

**Figure 6:**
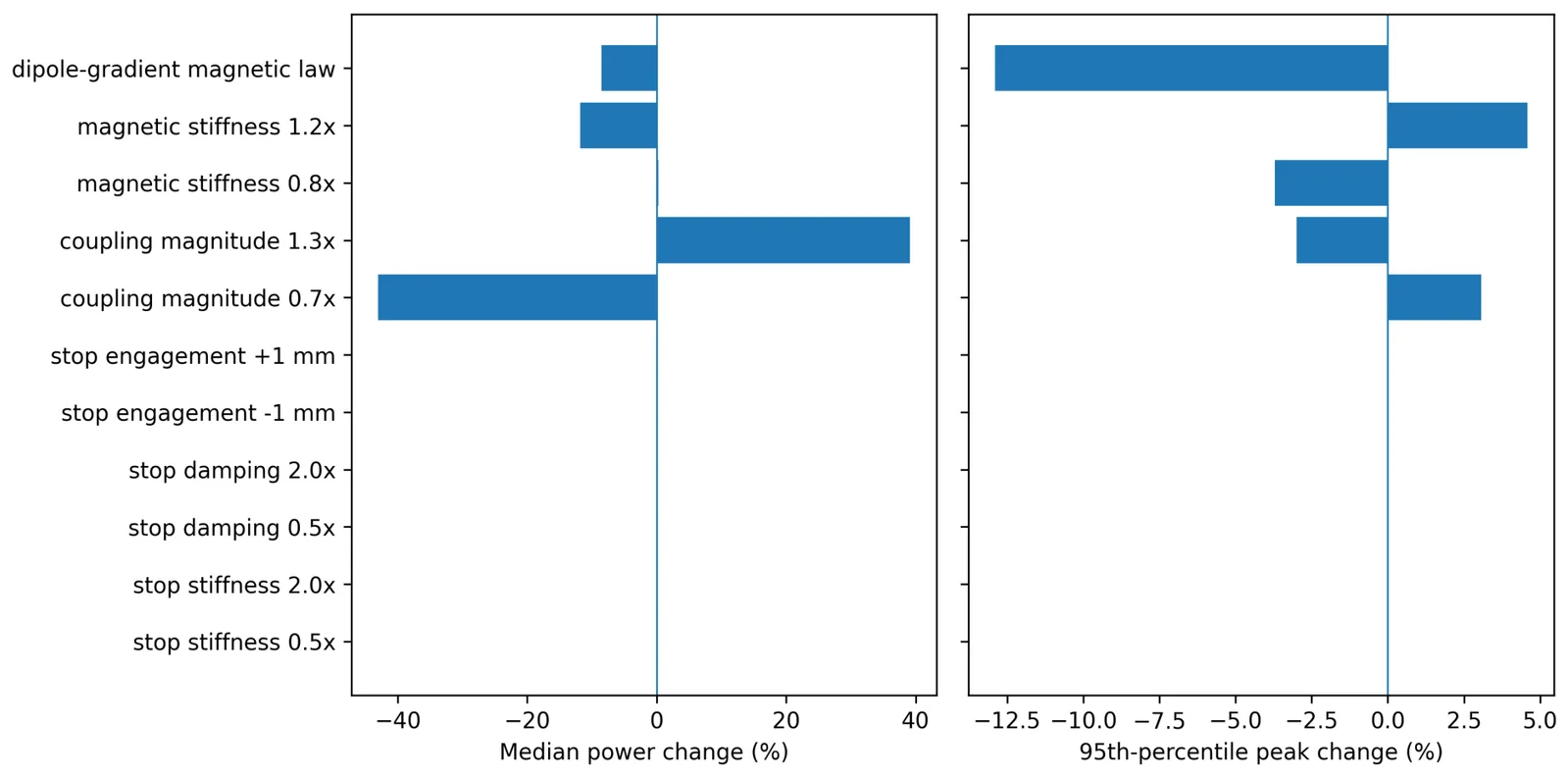
Sensitivity of median ideal load power and 95th-percentile peak displacement to individual parameter changes in 12 representative cases.

Figure 6 and Table 5 report one-at-a-time checks on 12 representative cases. The strongest effect is the assumed coupling scale. Reducing it by 30% lowers median ideal load power by 43.1%, while increasing it by 30% raises median power by 39.2%. This nonlinear response occurs because coupling changes both generated voltage and electromagnetic damping. A 20% increase in magnetic stiffness increases 95th-percentile peak displacement by 4.58% and lowers median power by 11.8%. Replacing the rational law with the dipole-gradient law lowers median power by 8.55% and 95th-percentile peak displacement by 12.90%.

Table 6 reports the full 30-candidate re-ranking. Candidate 600 remains first for the 1.3× coupling and both magnetic-stiffness cases. At 0.7× coupling, candidate 632 becomes first and candidate 600 becomes second. The highest-ranked geometry remains in the long family in every scenario. Thus the family-level choice is stable over the tested ranges, but the exact candidate is not invariant to the assumed coupling scale.

For the 40–45 s long-record window and sensor-*X* axis, changing the sensor-to-harvester offset from zero to ±20 mm changes ideal power from 3.962 mW to values between 3.469 and 4.419 mW. Peak displacement remains between 19.48 and 21.87 mm. All five offset cases remain design-safe. These values are a limited lever-arm sensitivity check, not a substitute for a measured installation geometry.

### 6.4. Usefulness Threshold

**Figure 7:**
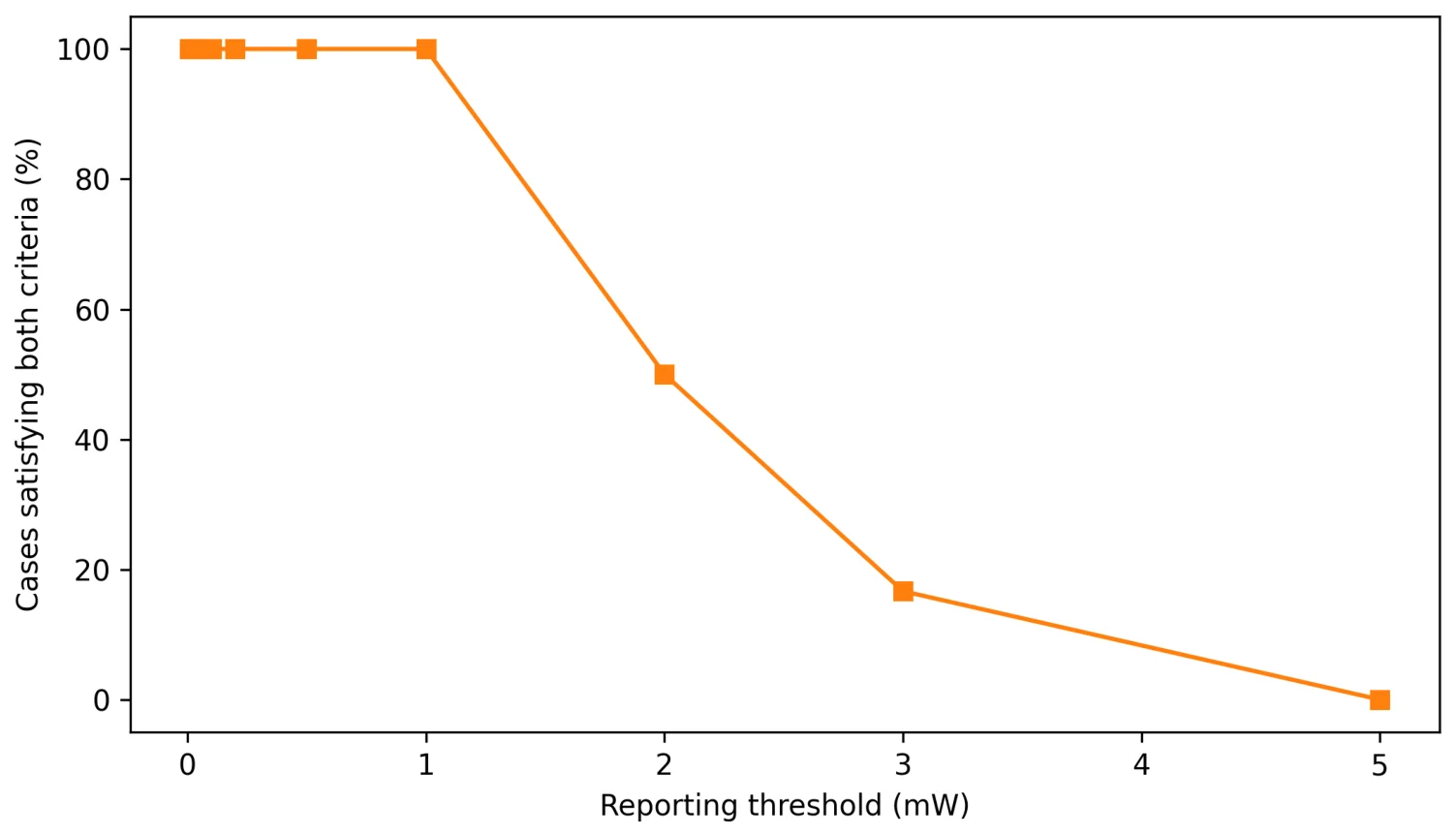
Fraction of selected-candidate cases that remain within both stroke limits and exceed each ideal load-power reporting threshold.

The 0.05 mW level is retained only as an example reporting threshold. It is not a general sensor requirement and is not used in the design ranking. Figure 7 shows the fraction of the selected candidate’s 72 cases above thresholds from 0.01 to 5 mW. All the cases exceed 1 mW; 50% exceed 2 mW; 16.7% exceed 3 mW; none exceed 5 mW.

At constant power, 0.05 mW corresponds to an ideal 20 s accumulation time for 1 mJ. With an assumed overall conversion efficiency of 30%, the corresponding time would be 66.7 s. This calculation only illustrates scale. Actual accumulation requires a rectifier, converter, storage element, start-up model, leakage model, and a specified sensor load.

### 6.5. Soft-Stop and Solver Checks

The selected candidate has a soft-stop start of 23.907 mm and a hard half-stroke of 30 mm, leaving 6.093 mm of numerical stop compression. The cubic elastic force reaches 8.47 N at the hard limit and stores 12.90 mJ over that compression. Dividing the hard-limit force by the 40.57 g moving mass gives a force-only acceleration scale of 208.8 $m\;s^{-2}$, equivalent to 21.3 times standard gravity. These values describe the numerical stop scale; they are not assigned to a tested bumper material.

The selected candidate does not contact the stop in the nominal 12-case subset; therefore, changing stop stiffness, damping, or engagement position has no effect in those nominal cases. To evaluate the stop model itself, the acceleration of the long-record 40–45 s sensor-*X* case is multiplied by 1.8. This is an explicit numerical stress input, not a measured additional gait trial. The nominal stop then engages for 2.6% of the samples, with peak displacement 25.68 mm and ideal power 5.84 mW. All the tested stop variations remain below the 30 mm hard half-stroke. Halving or doubling stop stiffness changes peak displacement by +0.12% and −0.22%. Halving or doubling stop damping changes it by +1.12% and −0.85%. Moving engagement by −1 or +1 mm changes it by −1.32% and +1.83%. The stress case exceeds the 24 mm design half-stroke by construction and is not counted in the Stage-2 performance statistics.

**Figure 8:**
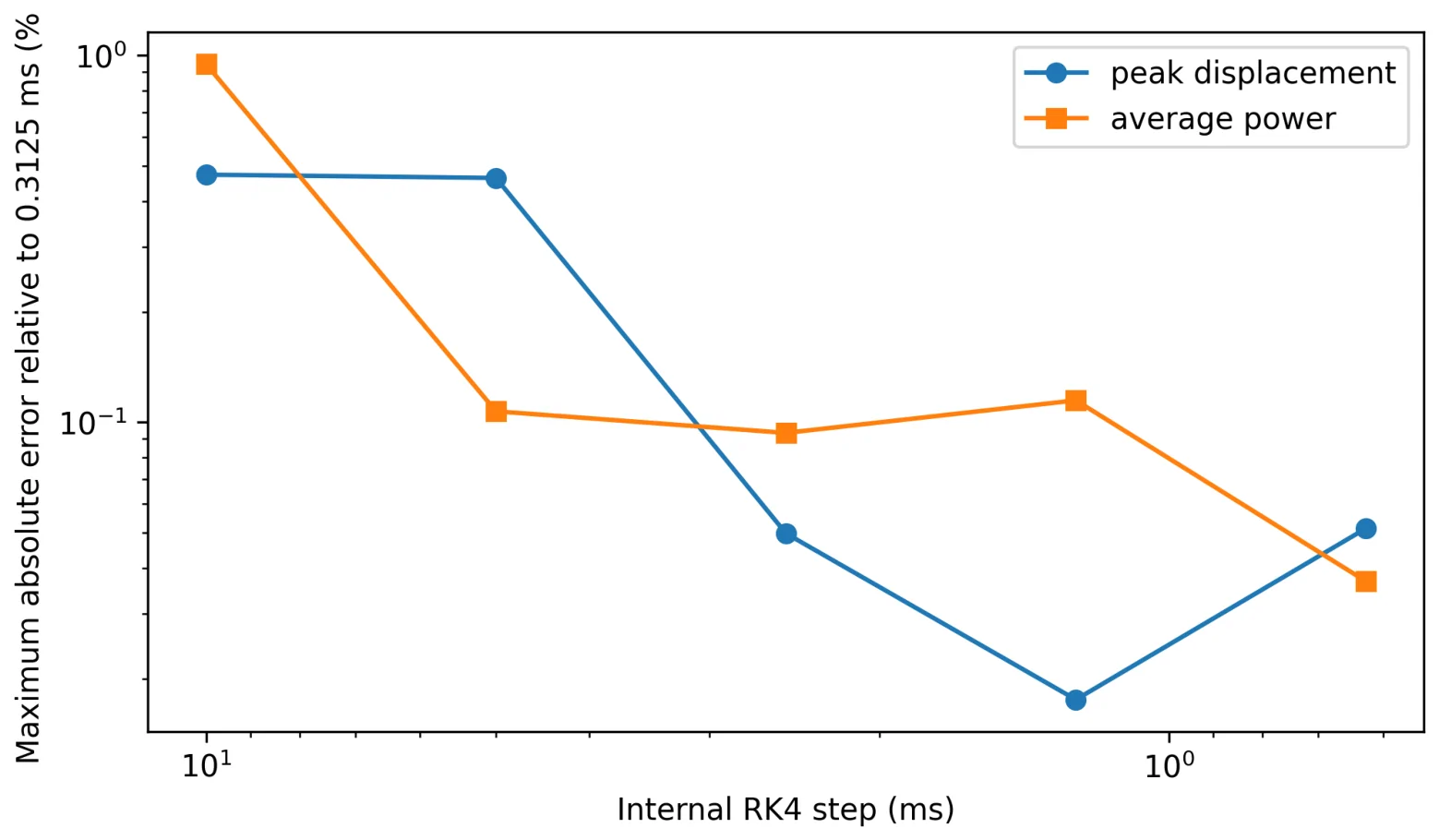
RK4 convergence for four nominal cases and one stop-engaging stress case. Errors are relative to 32 substeps per 10 ms input interval.

The dynamic equations are integrated with fixed-step RK4 between 100 Hz input samples, with linear interpolation of excitation within each interval. Convergence is checked for four nominal cases spanning both records, three axes, and both magnetic laws, plus the stop-engaging stress case. The reference uses 32 substeps per 10 ms input interval, corresponding to 0.3125 ms. At the four-substep setting used in Stage 2, the largest errors across the five cases are 0.0497% in peak displacement and 0.0935% in average power. Across the four nominal stop-free cases, the corresponding maxima are 0.000184% and 0.00194%. The stop-engaging case therefore controls the reported convergence bound. Figure 8 summarizes these convergence results.

## 7. Discussion

The input records contain measured foot-IMU motion, but they do not contain a calibrated transformation for the proposed harvester. The three design-screen axes and the 62-direction spherical grid are therefore installation scenarios rather than reconstructed hardware excitation. The direction sweep shows that the selected candidate remains inside the hard stroke over the sampled sphere, but the design-stroke margin is lost in one case for each of two opposite directions. A specific shoe or brace installation will require measured alignment and lever arm. Sensor-placement effects reported in gait-analysis studies support this requirement [18].

The magnetic and electrical formulations are internally consistent at the equation level. The two magnetic laws test force-shape dependence, and the flux-linkage model makes electromagnetic coupling position dependent. Section resistance, series connection, and polarity are explicit. The derivative, equilibrium-slope, and power-balance checks confirm that the equations are implemented consistently. They do not identify the physical force or coupling scale. Absolute power remains conditional on the assumed magnetic stiffness, coupling magnitude, and mechanical damping.

The selected design is the highest-ranked candidate among 720 evaluated candidates, not a global optimum. Full re-ranking under four parameter changes retains the long geometry family. Candidate 600 remains first for both magnetic-stiffness changes and increased coupling, while candidate 632 becomes first when coupling is reduced by 30%. This result separates a stable family-level conclusion from a coupling-sensitive candidate-level choice.

The stop parameters are expressed as engagement position, force at the hard limit, cubic exponent, and inward damping. This gives a physically interpretable numerical scale without assigning an untested material model. The nominal selected-candidate results are independent of stop parameters because the stop does not engage. The stress case and the multi-case convergence test quantify behavior when engagement occurs.

The selected 150 mm device remains difficult to integrate into normal footwear. Its 25.27 mm coil outer diameter and 40.57 g moving mass rule out a thin insole implementation. A realistic concept would require an external lateral rail or ankle–foot-brace enclosure with positive retention of the moving mass, guarded end stops, secure attachment, moisture protection, and tests for noise, comfort, impact durability, and effects on gait. The total housing, spring, collar, coil, fastener, and attachment mass is unknown. Consequently, this study supports a foot-worn module concept rather than a complete wearable product.

The electrical result is also limited to an ideal resistive load. Position-dependent coupling improves the mechanical–electrical consistency, but a practical interface will be affected by coil inductance, rectifier thresholds, converter input impedance, cold start, storage leakage, and duty-cycle demand [23]. No claim is made that the reported ideal power is delivered continuously to a sensor.

## 8. Limitations

The work has six main limitations.

1. Only two publicly available example foot-IMU records are evaluated. They are not a clinical cohort and do not support population statistics.
2. The sensor-to-harvester transformation is not calibrated. Explicit axis and offset cases bound some installation effects but do not reproduce a specific shoe or brace.
3. The magnetic force laws are analytical or surrogate normalized shapes. There is no magnetostatic finite-element model or measured force–displacement curve for the proposed collars.
4. The flux-linkage profile is analytical, but its absolute scale is normalized because magnet dimensions and remanence are unavailable. The ±30% study does not cover all possible coupling error.
5. The one-axis viscous model omits Coulomb friction, lateral motion, guide clearance, magnetic side forces, housing flexibility, wear, acoustic noise, and repeated-impact damage.
6. The electrical model omits inductance and power-management electronics. The reported power is ideal AC load-side power, not rectified, stored, or sensor-delivered power.

The next experimental step is to measure the magnetic force–displacement curve, open-circuit flux-linkage or voltage profile, guide friction and damping, soft-stop force and rebound, installed axis and lever arm, and capacitor charging through a specified interface circuit. These measurements can replace the normalized model scales without changing the finite-stroke computational framework.

## 9. Conclusions

A reproducible numerical screen was developed for a finite-stroke magnetic QZS moving-magnet harvester under public three-axis foot-IMU excitation. The analysis uses stated gravity and bias preprocessing, explicit mounting-axis vectors, rigid-body lever-arm terms, two magnetic force laws, and position-dependent flux linkage for series-connected coil sections with specified polarity.

Among 720 generated candidates, the highest-ranked nominal candidate is a 150 mm external foot-worn module with a 40.6 g moving mass, 30 mm hard half-stroke, and a two-section 1649-turn coil. It remains hard- and design-stroke-safe across the 72 design-screen combinations of windows, axes, and magnetic laws. Its conditional ideal load-side power has a 10th percentile of 1.38 mW and a median of 2.03 mW. A 62-direction post-selection sweep remains hard-stroke-safe in all 1488 cases, although two opposite directions each produce one design-stroke exceedance.

Absolute electromechanical coupling is the most influential tested uncertainty. Re-ranking all 30 Stage-2 candidates retains the long geometry family, but a 30% coupling reduction changes the highest-ranked candidate from 600 to 632. The reported mW values must therefore be treated as conditional numerical estimates until magnetic force, flux linkage, friction, stop behavior, and electrical conversion are measured. The supported engineering conclusion is that finite stroke, mounting orientation, coil polarity, position-dependent coupling, and uncertainty re-ranking should be included before prototype selection.

## Figures and Tables

**Table 1:** Representative related work and the scope of the present study.

Reference	Harvester or Scope	Evidence	Relation to the Present Study
Donelan et al. [9]	Knee-mounted biomechanical harvester	Experimental human walking	Demonstrates body-motion energy recovery at a larger joint scale.
Zhao and You [5]; Fan et al. [6]; Jeong et al. [7]	Shoe piezoelectric harvesters	Experimental prototypes	Use pressure or bending transduction rather than a guided magnetic QZS shuttle.
Wang et al. [8]	Sole-bending electromagnetic harvester	Experimental prototype	Uses sole bending to drive an electromagnetic generator; finite shuttle stroke is not the same design problem.
Digregorio and Redouté [17]	Wearable electromagnetic harvester	Device modeling and wearable context	Supports electromagnetic wearable design but does not address the same QZS and mounting reconstruction problem.
Wang et al. [15]; Cai et al. [16]	Low-frequency QZS electromagnetic harvesters	Numerical and/or laboratory vibration studies	Establish QZS electromagnetic concepts under prescribed vibration.
Küderle et al. [18]	Foot-IMU placement study	Experimental gait measurement	Shows that foot-sensor placement changes measured motion and motivates explicit mounting cases.
Present study	Finite-stroke magnetic QZS moving-magnet harvester	Reproducible numerical screen using two public raw foot-IMU records	Uses full three-axis preprocessing, explicit axis cases, two magnetic laws, position-dependent coupling, stated coil topology, and stop/solver sensitivity.

**Table 2:** Public IMU records and preprocessing checks.

Quantity	Short Record	Long Record
Unique raw samples	16,334	27,880
Duration (s)	41.618	70.732
Median raw sample rate (Hz)	398.319	398.546
Processed samples at 100 Hz	4163	7074
Static acceleration RMS after correction ($m\;s^{-2}$)	0.0146	0.0132
Static angular-rate RMS after correction ($\mathrm{rad}\;s^{-1}$)	0.00258	0.00260
AHRS accelerometer-feedback ignored fraction	0.287	0.414

**Table 3:** Common design-screen bounds. Uniform distributions are linear unless stated otherwise.

Parameter	Bound	Sampling
Moving mass	0.03–0.10 kg	uniform
Positive stiffness $k_{p}$	60–180 N/m	uniform
Magnetic ratio $k_{m}/k_{p}$	0.72–0.92	uniform
Magnetic length $x_{m}$	4–15 mm	uniform
Mechanical damping $c_{m}$	0.05–0.40 N s/m	log-uniform
Stop start $x_{s}/x_{\mathrm{hard}}$	0.68–0.84	uniform
Elastic stop force at hard limit	3–20 N	log-uniform
Stop damping	0.5–12 N s/m	log-uniform
Stop exponent	3	fixed
Load resistance	2–250 Ω	log-uniform
Wire catalogue	Cu0.20–Cu0.50	discrete uniform

**Table 4:** Parameters of the highest-ranked evaluated candidate.

Parameter	Value	Unit or Status
Device length	150.0	mm
Hard/design half-stroke	30.0/24.0	mm
Moving mass	40.57	g
Positive stiffness $k_{p}$	177.73	N/m
Magnetic stiffness $k_{m}$	129.46	N/m
Local effective stiffness $k_{p}-k_{m}$	48.27	N/m
Magnetic length $x_{m}$	10.82	mm
Mechanical damping	0.256	N s/m
Soft-stop start	23.91	mm
Elastic stop force at hard limit	8.47	N
Cubic stop coefficient	$3.745\times 10^{7}$	N/m^3^
Stop damping	2.321	N s/m
Turns/series sections	1649/2	+/− polarity
Wire	Cu0.25	0.25 mm bare diameter
Coil length/outer diameter	50.36/25.27	mm
Coil layers/mean radius	11/10.94	–/mm
Coil resistance/load resistance	39.87/43.33	Ω
Reference coupling scale	1.976	V s/m

**Table 5:** Selected uncertainty results relative to the nominal 12-case subset.

Case	Median Power Change (%)	95th-Percentile Peak Change (%)	Hard-Safe Fraction
Coupling scale 0.7×	−43.07	+3.06	1.00
Coupling scale 1.3×	+39.17	−2.99	1.00
Magnetic stiffness 0.8×	+0.28	−3.71	1.00
Magnetic stiffness 1.2×	−11.80	+4.58	1.00
Dipole-gradient force law	−8.55	−12.90	1.00

**Table 6:** Highest-ranked candidate after re-evaluating all 30 Stage-2 candidates under parameter changes.

Scenario	Candidate	Family	Hard Safe	Design Safe	$P_{10}$ (mW)	Median *P* (mW)	95th Peak (mm)
Nominal	600	Long	1.000	1.000	1.385	2.027	18.950
Coupling 0.7×	632	Long	1.000	1.000	0.841	1.302	19.011
Coupling 1.3×	600	Long	1.000	1.000	1.911	2.740	18.383
Magnetic stiffness 0.8×	600	Long	1.000	1.000	1.368	2.061	18.247
Magnetic stiffness 1.2×	600	Long	1.000	1.000	1.289	1.914	20.034

## Data Availability

The raw public IMU example files are available from the x-io Technologies Gait-Tracking repository cited in the manuscript and are redistributed in the source archive under the repository’s MIT license. The source archive also contains the processed records, candidate tables, result tables, figures, environment record, and analysis scripts needed to reproduce the reported numerical results.

## Supplementary Materials

The following supporting information can be downloaded at: https://www.mdpi.com/article/10.3390/mi17080892/s1. The Supplementary Materials contain the full preprocessing settings, mounting equations, magnetic and flux-linkage derivations, candidate bounds, window manifest, complete ranking details, orientation sweep, uncertainty re-ranking, equation-level checks, soft-stop stress results, and solver-convergence tables. The source archive contains the two public raw IMU files, complete license, processed data, all reported numerical outputs, and the Python scripts used for the analysis. The analyses were run with Python 3.13.5, NumPy 2.3.5, pandas 2.2.3, SciPy 1.17.0, Matplotlib 3.10.8, imufusion 1.3.1, and Numba 0.65.1.

## Abbreviations

AHRS	Attitude and heading reference system
IMU	Inertial measurement unit
QZS	Quasi-zero stiffness
RK4	Fourth-order Runge–Kutta method
RMS	Root mean square
