# Supplementary material for: Finite-Stroke Magnetic Quasi-Zero-Stiffness Electromagnetic Harvester for Foot-Worn Sensors: Reproducible Numerical Design Under Public Foot-IMU Excitation
Source: Micromachines (Basel). 2026 Jul 25;17(8):892. doi: 10.3390/mi17080892 (PMC13515382; doi:10.3390/mi17080892)
Supplement: Supplementary file 1 [file micromachines-17-00892-s001.zip › micromachines-4393728-supplementary.pdf]

Supplementary Materials: Finite-Stroke Magnetic Quasi-Zero-Stiffness Electromagnetic Harvester for Foot-Worn Sensors: Reproducible Numerical Design under Public Foot-IMU Excitation

Mohamed Hamdaoui

1. Scope and evidence boundary

The study is a numerical design screen. The source package contains the public raw foot-IMU records, their complete MIT license, preprocessing outputs, candidate parameters, case-level results, figures, and Python scripts. No magnetic finite-element model, physical force measurement, flux-linkage measurement, prototype test, rectifier simulation, or capacitor-charging experiment is included. The supplementary results must therefore be interpreted as conditional numerical results under the stated model parameters.

The two raw inputs are the `short_walk.csv` and `long_walk.csv` examples from the x-io Technologies Gait-Tracking repository. In the source archive, they are named `short_walk_public_imu.csv` and `long_walk_public_imu.csv`. The repository distributes the code and files under the MIT license. The source files provide time, three-axis gyroscope data in degrees per second, and three-axis accelerometer data in units of g.

2. IMU preprocessing

2.1. Interpolation, bias correction, and gravity removal

After duplicate timestamps are removed, each channel is linearly interpolated to a uniform 400 Hz grid. A six-axis Fusion attitude and heading reference system (AHRS) is run without a magnetometer. The settings are:

- coordinate convention: north–west–up;
- sample rate: 400 Hz;
- AHRS gain: 0.5;
- gyroscope range: 2000 $\deg s^{-1}$;
- acceleration-rejection threshold: 10°;
- magnetic rejection: disabled because no magnetometer is used;
- recovery trigger period: 5 s;
- gyroscope-bias stationary threshold: 3 $\deg s^{-1}$;
- gyroscope-bias stationary period: 3 s.

Let $\boldsymbol{f}_s$ be the measured accelerometer specific-force vector and $\widehat{\boldsymbol{g}}_s$ the AHRS gravity vector, both in the sensor frame and in units of g. The sensor-frame linear acceleration used in the model is

$$\boldsymbol{a}_s = g_0(\boldsymbol{f}_s - \widehat{\boldsymbol{g}}_s), \qquad g_0 = 9.80665\ \mathrm{m\,s^{-2}}. \tag{1}$$

This operation follows the Fusion definition of linear acceleration. The sign convention is therefore inherited from the public Fusion library. Gravity is not added a second time in the mechanical equation.

An eighth-order zero-phase Butterworth low-pass filter with a 14 Hz cut-off is applied to $\boldsymbol{a}_s$ and to angular rate $\boldsymbol{\omega}$. The 3.5–7.0 s interval is treated as static after the three-second bias-estimator start-up. Its mean residual acceleration and angular rate are subtracted. This step removes constant residual offsets only; it is not a six-position accelerometer calibration and does not estimate sensor scale or non-orthogonality.

Angular acceleration is calculated as

$$\boldsymbol{\alpha}(t) = \frac{\mathrm{d}\boldsymbol{\omega}}{\mathrm{d}t} \tag{2}$$

using a centered numerical gradient with second-order edge treatment, followed by the same zero-phase 14 Hz low-pass filter. All processed vectors are then resampled to 100 Hz for the dynamic simulation.

2.2. Numerical preprocessing outputs

Table S1 lists the complete residual offsets. The final gyroscope-bias values are returned by the Fusion bias estimator. The residual values are the means removed over 3.5–7.0 s after filtering.

Table S1. Preprocessing outputs for the two public records.

Quantity	Short record	Long record
Unique raw rows	16,334	27,880
Duration (s)	41.61802959	70.73208332
Median raw sample rate (Hz)	398.319093	398.546104
Processed rows at 100 Hz	4,163	7,074
Final Fusion bias, X ($\mathrm{deg\,s^{-1}}$)	−0.0502647	−0.0124569
Final Fusion bias, Y ($\mathrm{deg\,s^{-1}}$)	−0.0188356	+0.0159428
Final Fusion bias, Z ($\mathrm{deg\,s^{-1}}$)	−0.0540072	−0.0083986
Residual acceleration removed, X ($\mathrm{m\,s^{-2}}$)	−0.0243009	+0.0433346
Residual acceleration removed, Y ($\mathrm{m\,s^{-2}}$)	+0.0301792	−0.0137226
Residual acceleration removed, Z ($\mathrm{m\,s^{-2}}$)	−0.0169328	−0.0422182
Residual angular rate removed, X ($\mathrm{rad\,s^{-1}}$)	−0.0011635	−0.0002166
Residual angular rate removed, Y ($\mathrm{rad\,s^{-1}}$)	−0.0021657	+0.0010693
Residual angular rate removed, Z ($\mathrm{rad\,s^{-1}}$)	−0.0011820	−0.0008687
Static acceleration RMS after correction ($\mathrm{m\,s^{-2}}$)	0.0146235	0.0132065
Static angular-rate RMS after correction ($\mathrm{rad\,s^{-1}}$)	0.0025804	0.0026017
AHRS accelerometer-feedback ignored fraction	0.287293	0.413910

The accelerometer-feedback ignored fraction is an AHRS algorithm status flag. It means that accelerometer feedback was not used to correct orientation during strongly dynamic periods. It does not mean that those acceleration samples were removed from the harvester excitation.

3. Sensor-to-harvester transformation

3.1. General rotation-matrix form

Let $\mathcal{S}$ be the sensor frame and $\mathcal{H}$ a harvester frame whose first axis is the shuttle axis. If $\boldsymbol{R}_{HS}$ maps a vector from $\mathcal{S}$ to $\mathcal{H}$, then the axial input is the first component of

$${}^{H}\boldsymbol{a}_{H} = \boldsymbol{R}_{HS}\left[{}^{S}\boldsymbol{a}_{S} + {}^{S}\boldsymbol{\alpha} \times {}^{S}\boldsymbol{r}_{H/S} + {}^{S}\boldsymbol{\omega} \times \left({}^{S}\boldsymbol{\omega} \times {}^{S}\boldsymbol{r}_{H/S}\right)\right]. \tag{3}$$

Equivalently, if $\boldsymbol{e}_h$ is the first row of $\boldsymbol{R}_{HS}$ written as a column vector in $\mathcal{S}$,

$$a_b = \boldsymbol{e}_h^{\mathsf{T}}[\boldsymbol{a}_S + \boldsymbol{\alpha} \times \boldsymbol{r}_{H/S} + \boldsymbol{\omega} \times (\boldsymbol{\omega} \times \boldsymbol{r}_{H/S})]. \tag{4}$$

Only the first row of $\boldsymbol{R}_{HS}$ is required by the one-axis mechanical model. The full matrix would be required for a multiaxis shuttle model.

3.2. Evaluated axis and offset cases

No calibrated $\boldsymbol{R}_{HS}$ is available. The evaluated axis vectors are therefore

$$\boldsymbol{e}_X = [1,0,0]^\top, \quad (5)$$
$$\boldsymbol{e}_Y = [0,1,0]^\top, \quad (6)$$
$$\boldsymbol{e}_{XZ30} = [\cos 30^\circ, 0, \sin 30^\circ]^\top. \quad (7)$$

These are scenario definitions, not estimates of the actual public sensor placement. The main screen uses $\boldsymbol{r}_{H/S} = \boldsymbol{0}$. The offset check uses $\boldsymbol{r}_{H/S} = \pm 20$ mm along the sensor axes as listed in Table S2.

After candidate selection, a deterministic spherical grid is used to assess additional axis directions:

$$\boldsymbol{e}_h(\beta, \psi) = [\sin\beta\cos\psi,\ \sin\beta\sin\psi,\ \cos\beta]^\top. \quad (8)$$

The polar angles are $0^\circ, 30^\circ, 60^\circ, 90^\circ, 120^\circ, 150^\circ, 180^\circ$. At the five intermediate polar angles, the azimuth is sampled from 0° to 330° in 30° steps. One direction is retained at each pole. The resulting 62 unit vectors are evaluated for all 12 windows and both magnetic laws, giving $62 \times 12 \times 2 = 1488$ cases. This grid is not used to re-optimize the candidate.

Table S2. Lever-arm sensitivity for the long-record 40–45 s sensor-X case.

Offset	Peak displacement (mm)	Ideal load power (mW)	RMS load voltage (V)
Co-located	20.721	3.962	0.4140
+20 mm sensor X	20.666	3.875	0.4094
+20 mm sensor Y	20.396	3.878	0.4097
+20 mm sensor Z	21.870	4.419	0.4373
−20 mm sensor Z	19.479	3.469	0.3873

All offset cases remain below the 24 mm design half-stroke. This check covers only one record window, one axis, and a 20 mm offset magnitude.

4. Magnetic force models

4.1. Rational force law

The rational law is

$$F_m^{(R)}(x) = -\frac{k_m x}{1 + (x/x_m)^2}. \quad (9)$$

Its derivative is

$$\frac{\mathrm{d}F_m^{(R)}}{\mathrm{d}x} = -k_m \frac{1 - (x/x_m)^2}{[1 + (x/x_m)^2]^2}, \quad (10)$$

so its equilibrium slope is $-k_m$ and the force magnitude reaches a maximum at $|x| = x_m$.

4.2. Dipole-gradient force law

For a simplified dipole interaction with fixed transverse separation x_m, a potential shape may be written as

$$U(x) = \frac{C}{(x_m^2 + x^2)^{3/2}}. \quad (11)$$

Its axial gradient is proportional to

$$-\frac{\mathrm{d}U}{\mathrm{d}x} \propto \frac{x}{(x_m^2 + x^2)^{5/2}}. \quad (12)$$

Choosing the polarity that gives negative stiffness and normalizing the local slope produces

$$F_m^{(D)}(x) = -\frac{k_m x}{[1+(x/x_m)^2]^{5/2}}. \tag{13}$$

This law is an analytical model-form check. The proposed magnetic collar geometry is not represented explicitly.

4.3. Separate ranking by force law

Table S3 reports the selected candidate under each force law. Candidate 600 ranks first in both separately ranked data sets.

Table S3. Selected candidate results separated by magnetic force law.

Law	Cases	Hard safe	Design safe	P_{10} (mW)	Median P (mW)	95th peak (mm)
Rational	36	1.00	1.00	1.555	2.106	20.040
Dipole-gradient	36	1.00	1.00	1.302	1.984	17.139

5. Position-dependent flux linkage

5.1. Circular-loop expression

For a point magnetic dipole on the axis of a circular loop of radius a, the axial magnetic flux through one loop has the spatial form

$$\phi(z) = C_m \frac{a^2}{2(a^2+z^2)^{3/2}}, \tag{14}$$

where C_m contains the magnetic constant and dipole moment. Differentiation with respect to magnet displacement x for a loop at coordinate z_l gives

$$\frac{\mathrm{d}\phi(z_l - x)}{\mathrm{d}x} = 3C_m \frac{a^2(z_l - x)}{2[a^2+(z_l - x)^2]^{5/2}}. \tag{15}$$

The code integrates this expression by averaging 81 uniformly spaced loop positions within each coil section.

5.2. Series sections and resistance

For n_s sections,

$$\lambda(x) = \sum_{j=1}^{n_s} s_j \lambda_j(x), \qquad R_c = \sum_{j=1}^{n_s} R_{c,j}. \tag{16}$$

The sign s_j represents winding direction or electrical connection polarity. Total turns are divided as evenly as possible among sections. The selected candidate has 1649 turns, so its two sections contain 825 and 824 turns.

The selected polarity is $+/-$. Table S4 compares the unscaled RMS coupling over the design stroke. The absolute raw values contain the omitted dipole scale and are not physical coupling values; their ratio quantifies cancellation.

Table S4. Two-section winding-polarity comparison for the selected candidate.

Section signs	Unscaled RMS coupling	Ratio to best
$+/-$	1,419,942.25	1.000
$+/+$	671,963.42	0.473

5.3. Energy consistency for the resistive model

With $\kappa(x) = \mathrm{d}\lambda/\mathrm{d}x$, Faraday's law gives $e = -\kappa\dot{x}$. For total resistance $R_c + R_L$,

$$i = -\frac{\kappa\dot{x}}{R_c + R_L}. \quad (17)$$

The electromagnetic generalized force is $F_e = \kappa i$, which gives

$$F_e\dot{x} = -\frac{\kappa^2\dot{x}^2}{R_c + R_L} \leq 0. \quad (18)$$

The electrical reaction therefore removes mechanical energy. The total resistive dissipation is

$$i^2(R_c + R_L) = \frac{\kappa^2\dot{x}^2}{R_c + R_L}, \quad (19)$$

which equals the mechanical power removed in magnitude. Load power is the fraction $R_L/(R_c + R_L)$ of this total dissipation.

5.4. Equation-level implementation checks

Table S5 gives three numerical checks on the implemented equations. The flux-linkage derivative check uses a 10,001-point displacement grid with 7.5 μm spacing. The magnetic-slope check uses a centered derivative at equilibrium. The energy-balance check uses the long-record 40–45 s, X–Z 30°, rational-law case.

Table S5. Equation-level implementation checks.

Check	Result
Flux derivative	Maximum finite-difference error $= 2.6 \times 10^{-5}$% of peak coupling; RMS error $= 1.8 \times 10^{-5}$% of RMS coupling.
Magnetic slope	Rational and dipole-gradient numerical slopes agree with -129.460176 N/m to within 2.2×10^{-10}%.
Electromagnetic power balance	Maximum instantaneous mismatch $= 2.78 \times 10^{-17}$ W, equal to 2.35×10^{-14}% of peak total electrical power. Mean total resistive power is 5.074 mW, of which 2.643 mW reaches the load.

These checks establish numerical consistency with the stated equations. They do not provide experimental or finite-element validation of the physical force and coupling scales.

6. Candidate generation and design-screen procedure

6.1. Geometry-dependent bounds

Table S6 lists the family-specific values. Total coil length is divided equally among the selected number of sections.

Table S6. Geometry-family bounds.

Family	Length (mm)	Hard/design half-stroke (mm)	Turns	Coil length (mm)	Sections
Compact	90	15.0/12.0	200–1100	12–30	1–2
Medium	120	22.0/17.6	300–2200	18–45	1–3
Long	150	30.0/24.0	400–3000	25–60	1–3

The common ranges are listed in the main paper. Mechanical damping, stop force, stop damping, and load resistance are sampled log-uniformly because their ranges span multiplicative scales. All other continuous parameters are sampled uniformly. The wire catalogue is

Wire	Bare copper diameter (mm)	Insulated diameter (mm)
Cu0.20	0.20	0.23
Cu0.25	0.25	0.285
Cu0.32	0.32	0.36
Cu0.40	0.40	0.45
Cu0.50	0.50	0.56

The fixed tube and winding quantities are: tube outer diameter 18 mm, radial clearance 0.5 mm, maximum coil outer diameter 26 mm, turn pitch factor 1.08, copper resistivity $1.724 \times 10^{-8}\ \Omega\,\mathrm{m}$, and lead length 0.15 m.

6.2. Window manifest

Table S7 lists every Stage-2 window after 100 Hz preprocessing. Acceleration RMS is the three-axis vector-norm RMS before harvester-axis projection.

Table S7. Five-second input windows used in Stage 2.

Window	Start (s)	End (s)	Acceleration RMS ($\mathrm{m\,s^{-2}}$)	Angular-rate RMS ($\mathrm{rad\,s^{-1}}$)
short_15_20	15	20	14.508	4.513
short_20_25	20	25	13.433	4.256
short_25_30	25	30	15.724	4.921
short_30_35	30	35	12.314	3.632
long_15_20	15	20	13.622	4.224
long_20_25	20	25	13.548	4.144
long_25_30	25	30	14.512	4.221
long_30_35	30	35	14.604	4.348
long_35_40	35	40	15.314	4.373
long_40_45	40	45	15.596	4.581
long_45_50	45	50	13.832	4.263
long_50_55	50	55	15.229	4.354

Stage 1 uses `short_20_25`, `short_25_30`, `long_25_30`, and `long_40_45`. Stage 2 uses all windows in Table S7.

6.3. Algorithm specification

The algorithm is:

1. Initialize NumPy's default pseudorandom generator with seed 4,393,728.
2. For each family, sample parameters until 240 winding-feasible candidates have been generated.
3. Determine winding layers, outer diameter, mean radius, wire length, resistance, and reference coupling scale from geometry.
4. Enumerate section polarity combinations and retain the combination with the highest unscaled RMS coupling over the design stroke.
5. Stage 1: evaluate all 720 candidates on four windows with sensor-X axis, rational magnetic law, and three RK4 substeps per 10 ms interval.
6. Rank candidates within each family by hard safety, design safety, 10th-percentile power, median power, then 95th-percentile displacement. Retain ten per family.
7. Stage 2: evaluate 30 candidates on 12 windows, three axes, and two magnetic laws, with four RK4 substeps per 10 ms interval.
8. Apply the same lexicographic ranking across the 30 candidates. Report the first candidate as the highest-ranked evaluated candidate.

The total number of Stage-1 simulations is $720 \times 4 = 2880$. The total number of Stage-2 simulations is $30 \times 12 \times 3 \times 2 = 2160$.

6.4. Full-candidate uncertainty re-ranking

All 30 Stage-2 candidates are evaluated again under four global parameter changes: coupling scale 0.7× and 1.3×, and magnetic stiffness 0.8× and 1.2×. Each candidate is run over the same 72 cases, and the same lexicographic ranking is applied. The additional simulation count is $30 \times 72 \times 4 = 8640$. Candidate geometry, load resistance, coil connection, and all other sampled parameters remain fixed within each scenario.

6.5. Candidate-count stability

Table S8 repeats the Stage-1 selection on prefixes of the fixed candidate sequence. The long-family candidate stabilizes at candidate 600 once 120 long-family candidates are included. This is a repeatability check on the fixed sample sequence, not a statistical convergence proof.

Table S8. Selected Stage-1 candidate identifier versus generated candidates per family.

Family	60	120	180	240
Compact	40	67	176	176
Medium	260	309	420	420
Long	498	600	600	600

7. Additional results

7.1. Mounting-axis results

Table S9 groups the selected candidate's Stage-2 results by axis. Each row contains 12 windows and two magnetic laws.

Table S9. Selected-candidate results by explicit harvester-axis case.

Axis	Cases	P_{10} (mW)	Median P (mW)	Median peak (mm)	Maximum peak (mm)	Design safe
Sensor X	24	2.011	3.005	16.627	21.222	1.00
Sensor Y	24	1.166	1.644	11.096	13.724	1.00
X–Z, 30°	24	1.565	1.911	16.005	18.895	1.00

7.2. Full-sphere orientation results

Table S10 summarizes the 62-direction post-selection sweep. A direction is listed as fully design-safe only if all 24 combinations of windows and magnetic laws remain within the 24 mm design half-stroke.

Table S10. Summary of the 62-direction orientation sweep.

Quantity	Result
Directions	62
Cases per direction	24
Total cases	1488
Hard-stroke-safe cases	1488/1488
Directions with all 24 cases design-safe	60/62
Minimum design-safe fraction for a direction	23/24 (0.9583)
Direction-level median-power range	0.952–3.077 mW
Maximum peak displacement	24.151 mm

The two directions with one design-stroke exceedance are opposite directions on the spherical grid. This symmetry is expected because reversing the axis sign changes the signs of projected input and relative displacement but not the displacement magnitude used for the stroke metric.

7.3. Highest-ranked candidate in each family

Table S11. Stage-2 family comparison.

Family	Candidate	Hard safe	Design safe	P_{10} (mW)	Median P (mW)	95th peak (mm)
Compact	205	1.00	0.528	0.0658	0.1129	12.827
Medium	309	1.00	1.000	0.3435	0.4826	17.147
Long	600	1.00	1.000	1.3846	2.0268	18.950

7.4. Uncertainty re-ranking

Table S12 gives the first-ranked candidate under the nominal and four global-parameter scenarios. The full ranked table for every scenario is included in the source archive.

Table S12. Highest-ranked candidate after re-evaluating all 30 Stage-2 candidates.

Scenario	Candidate	Family	Hard safe	Design safe	P_{10} (mW)	Median P (mW)	95th peak (mm)
Nominal	600	Long	1.000	1.000	1.385	2.027	18.950
Coupling 0.7×	632	Long	1.000	1.000	0.841	1.302	19.011
Coupling 1.3×	600	Long	1.000	1.000	1.911	2.740	18.383
Magnetic stiffness 0.8×	600	Long	1.000	1.000	1.368	2.061	18.247
Magnetic stiffness 1.2×	600	Long	1.000	1.000	1.289	1.914	20.034

Candidate 600 remains first in three of the four uncertainty scenarios. Candidate 632 becomes first at 0.7× coupling, while candidate 600 becomes second. The long family remains first in all tested scenarios.

7.5. Threshold sweep

Table S13. Selected-candidate reporting-threshold sweep. The threshold is not part of the ranking.

Threshold (mW)	Cases above (fraction)	Ideal time for 1 mJ (s)	Time at 30% efficiency (s)
0.01	1.000	100.0	333.3
0.025	1.000	40.0	133.3
0.05	1.000	20.0	66.7
0.10	1.000	10.0	33.3
0.20	1.000	5.0	16.7
0.50	1.000	2.0	6.7
1.00	1.000	1.0	3.3
2.00	0.500	0.5	1.7
3.00	0.167	0.333	1.111
5.00	0.000	0.2	0.667

The accumulation times are calculated from $t = E/P$. Values at 30% are not circuit simulations.

7.6. Nominal parameter sensitivity

Table S14 gives the complete 12-case one-at-a-time sensitivity. Stop changes have no effect because the selected candidate does not contact the stop in this subset.

Table S14. Complete nominal one-at-a-time sensitivity results.

Case	Hard safe	Design safe	Median power (mW)	Median peak (mm)	Power change (%)	95th-peak change (%)
Nominal	1.00	1.00	2.15345	17.3169	0.000	0.000
Stop stiffness 0.5×	1.00	1.00	2.15345	17.3169	0.000	0.000
Stop stiffness 2.0×	1.00	1.00	2.15345	17.3169	0.000	0.000
Stop damping 0.5×	1.00	1.00	2.15345	17.3169	0.000	0.000
Stop damping 2.0×	1.00	1.00	2.15345	17.3169	0.000	0.000
Stop engagement −1 mm	1.00	1.00	2.15345	17.3169	0.000	0.000
Stop engagement +1 mm	1.00	1.00	2.15345	17.3169	0.000	0.000
Coupling 0.7×	1.00	1.00	1.22605	17.6987	−43.066	+3.060
Coupling 1.3×	1.00	1.00	2.99685	16.9059	+39.165	−2.994
Magnetic stiffness 0.8×	1.00	1.00	2.15940	16.1930	+0.277	−3.712
Magnetic stiffness 1.2×	1.00	1.00	1.89925	17.7373	−11.804	+4.580
Dipole-gradient force law	1.00	1.00	1.96933	13.7915	−8.550	−12.895

7.7. Soft-stop physical scale and stress test

For the selected candidate, the stop begins at 23.907 mm and the hard half-stroke is 30 mm. The available numerical stop compression is therefore 6.093 mm. At the hard limit, the cubic elastic force is 8.472 N, and the stored elastic energy is

$$E_s = \int_0^{\delta_h} k_s \delta^3 \, \mathrm{d}\delta = \frac{F_{s,\mathrm{hard}} \delta_h}{4} = 12.904 \text{ mJ}. \quad (20)$$

The ratio of hard-limit force to moving mass is 208.8 $\mathrm{m\,s^{-2}}$ (21.3 g). These are numerical design values, not measured properties of a specified elastomer or bumper.

The input acceleration of the long-record 40–45 s sensor-X case is multiplied by 1.8 solely to produce controlled stop engagement. Table S15 lists the results.

Table S15. Soft-stop sensitivity under the explicit 1.8× acceleration stress case.

Case	Peak (mm)	Power (mW)	Contact fraction	Peak change (%)	Power change (%)
Nominal stop	25.682	5.843	0.026	0.000	0.000
Stop stiffness 0.5×	25.712	5.879	0.026	+0.117	+0.614
Stop stiffness 2.0×	25.625	5.810	0.026	−0.224	−0.572
Stop damping 0.5×	25.971	5.860	0.026	+1.124	+0.291
Stop damping 2.0×	25.463	5.807	0.024	−0.852	−0.625
Engagement −1 mm	25.344	5.775	0.036	−1.317	−1.174
Engagement +1 mm	26.152	5.918	0.018	+1.829	+1.285

All stress cases remain inside the hard stroke and outside the design stroke.

7.8. RK4 convergence

The 100 Hz input samples are linearly interpolated during fixed-step RK4 integration. Four nominal cases span both input records, all three design-screen axes, and both magnetic laws. A fifth case uses the 1.8× stop-engaging stress input. The reference uses 32 substeps per 10 ms input interval (0.3125 ms). Table S16 gives the errors at the four-substep setting used in Stage 2.

Table S16. RK4 errors at four substeps per 10 ms relative to the 32-substep reference.

Case	Internal step (ms)	Peak-displacement error (%)	Average-power error (%)
Short 20–25 s, sensor X, rational law	2.5	0.000048	0.000906
Short 30–35 s, sensor Y, dipole-gradient law	2.5	0.000113	0.000755
Long 40–45 s, X–Z 30°, rational law	2.5	0.000019	0.000397
Long 45–50 s, sensor X, dipole-gradient law	2.5	0.000184	0.001941
Long 40–45 s, X–Z 30°, rational law, 1.8× stress	2.5	0.049742	0.093450

The largest errors at the production setting are therefore 0.0497% in peak displacement and 0.0935% in average power. The stop-engaging stress case controls both maxima.

8. Software environment and archive contents

The analysis was executed with Python 3.13.5, NumPy 2.3.5, pandas 2.2.3, SciPy 1.17.0, Matplotlib 3.10.8, imufusion 1.3.1, and Numba as recorded in `data/study_`

environment.json. The main script is code/reproducible_design_analysis.py. Additional orientation, re-ranking, equation-check, and multi-case convergence analyses are in code/extended_robustness_checks.py. The article-figure script is code/make_article_figures.py.

The principal output files are:

- study_preprocessing_summary.csv and study_preprocessed_imu.csv;
- study_window_manifest.csv;
- study_candidate_parameters.csv and study_optimization_bounds.csv;
- study_stage1_candidates.csv, study_stage2_cases.csv, and study_stage2_ranked.csv;
- study_selected_design.csv and study_model_specific_ranking.csv;
- study_orientation_sweep.csv and study_uncertainty_reranking.csv;
- study_parameter_sensitivity.csv and study_stop_stress_sensitivity.csv;
- study_solver_convergence_multicase.csv;
- study_flux_derivative_check.csv, study_magnetic_slope_check.csv, and study_electromagnetic_energy_balance.csv;
- study_threshold_sweep.csv, study_coil_polarity_comparison.csv, and study_lever_arm_sensitivity.csv.

9. Measurements needed to replace normalized assumptions

The following measurements are required before the numerical mW values can be treated as hardware predictions:

1. axial magnetic force versus shuttle displacement for the installed collars, including alignment tolerance;
2. open-circuit flux linkage or induced voltage versus shuttle position and speed for each coil section;
3. coil inductance and resistance at the relevant frequency range;
4. guide friction, viscous damping, and lateral side load;
5. soft-stop force, hysteresis, rebound, and durability;
6. calibrated sensor-to-harvester rotation and lever arm in the selected shoe or brace;
7. rectified capacitor charging through a specified power-management circuit.

These measurements can be inserted into the released code by replacing the normalized force and coupling arrays and by extending the electrical state equation.
